# Graded Carbohydrate Ingestion up to 120 g·h^−1^ Attenuates the Reduction in Critical Power Following 3 h of Moderate‐Intensity Exercise in a Dose‐Dependent Manner

**DOI:** 10.1111/sms.70326

**Published:** 2026-06-19

**Authors:** Bernardo R. Norte, Mollie M. Slinn, Kelsie O. Johnson, Elizabeth Mahon, Sam O. Shepherd, Juliette A. Strauss, Julien B. Louis

**Affiliations:** ^1^ Research Institute for Sport and Exercise Sciences (RISES) Liverpool John Moores University Liverpool UK; ^2^ Precision Fuel & Hydration, Ltd Christchurch UK

**Keywords:** durability, exercise intensity domains, exercise metabolism, fatigue, performance, physiological resilience, physiology, power‐duration relationship

## Abstract

This study examined the effects of graded carbohydrate (CHO) ingestion at rates of 0 (water only), 60 and 120 g·h^−1^ (1:0.8 ratio of maltodextrin‐to‐fructose) on changes in critical power following 3‐h of moderate‐intensity cycling in endurance‐trained cyclists/triathletes (*n* = 16; V̇O_2max_ 51.8 ± 6.8 mL·kg^−1^·min^−1^). Following a standardized 24‐h CHO loading protocol (8 g·kg^−1^) and pre‐exercise meal (2 g·kg^−1^), participants completed a 3‐min critical power (CP) test, with end‐test power (EP) and work done above EP used as estimates of CP and W*′*, respectively, in a non‐fatigued state and after 3‐h of cycling at 95% gas exchange threshold (‘fatigued’ state). CP was significantly reduced (*p* < 0.001) after the 3‐h exercise in all experimental conditions (water: 236 ± 30 W; 60 g·h^−1^: 257 ± 28 W; 120 g·h^−1^: 266 ± 29 W) compared to the non‐fatigued state (277 ± 27 W). However, this reduction in CP was attenuated with increasing CHO intake during exercise in a dose‐dependent manner, such that CP after 3‐h was greater in the 120 g·h^−1^ vs. 60 g·h^−1^ vs. water condition (*p* < 0.05). W*′* declined over time, with no differences between fatigued conditions (*p* > 0.05). Mean whole‐body CHO oxidation rates were significantly higher (*p* < 0.001) with increasing CHO intake (water: 1.84 ± 0.28; 60 g·h^−1^: 2.16 ± 0.15; 120 g·h^−1^: 2.31 ± 0.14 g·min^−1^). These data suggest that CHO ingestion at 120 g·h^−1^ limits the reduction in CP following prolonged moderate‐intensity cycling, with no effect on W*′*. These findings demonstrate that the boundary between heavy‐ and severe‐intensity exercise shifts under fatigue, with CHO availability possibly acting as a key modulator of endurance durability.

## 1 Introduction

Endurance performance has traditionally been determined by maximal oxygen uptake (V̇O_2max_), fractional utilization of lactate threshold, and exercise economy [1]. While these metrics provide valuable insights into an athlete's physiological profile in a rested state, they fail to account for the dynamic changes in physiological responses during prolonged endurance exercise [2]. Indeed, prolonged exercise is accompanied by numerous physiological perturbations, including deteriorations in exercise economy [3], elevated core and muscle temperature [4], loss of neuromuscular function [5], and depletion of endogenous fuel stores [6]. The ability to withstand the functional decline in physiological characteristics over time has been termed durability or physiological resilience [2, 7]. While these terms are used interchangeably in the literature, durability more precisely refers to changes in external power output or speed at anchor points in the power‐duration curve (e.g., lactate threshold, critical power [CP]), whereas physiological resilience describes the robustness of physiological determinants of performance (e.g., maximal oxygen uptake [V̇O_2max_], exercise economy/efficiency) and changes in underlying physiology in the face of accumulating work done [8].

Durability has emerged as a central theme in contemporary endurance research [2, 7, 9, 10]. Field‐based studies in professional road cycling demonstrate that sustaining high mean maximal power outputs under increasing levels of accumulated work distinguishes WorldTour from ProTeam riders [11]. Laboratory‐based studies have examined durability by anchoring fatiguing protocols around exercise intensity domains, whereby athletes exercise at a fixed intensity within the moderate or heavy domains. Using exercise intensity domains to establish mechanisms of fatigue resistance is worthwhile, as metabolic perturbations and loss of muscle homeostasis are differentially manifested across domains [12]. Notably, increasing evidence indicates that as mechanical work accumulates, the exercise intensity at which these domain transitions occur shifts downward [13, 14], meaning these physiological boundaries do not remain static during prolonged exercise.

CP demarcates the boundary between heavy‐ and severe‐intensity exercise [15], above which a finite amount of work can be performed, defined as the curvature constant of the power‐duration relationship, known as W*′* [15]. Fatigue‐induced reductions in CP may compress the heavy‐intensity domain, causing initially moderate or heavy exercise intensities to inevitably drift toward higher intensity domains and compromise subsequent severe‐intensity exercise. However, the temporal changes of these intensity domain transitions under fatigue remain poorly understood.

The degradation of physiological profiling characteristics following prolonged exercise has been examined using protocols anchored around the first ventilatory threshold, with fatiguing tests focusing on the durability of the moderate‐to‐heavy transition [13, 14, 16, 17, 18] and, in some instances, on severe‐intensity performance [17, 18]. Conversely, only one study [19] has assessed the durability of the heavy‐to‐severe domain transition (i.e., CP) with a fatiguing exercise protocol performed in the heavy domain. In this study, Clark et al. [19] showed that carbohydrate (CHO) ingestion negated a ~9% reduction in CP following 2 h of heavy‐intensity cycling, suggesting durability may be linked to CHO availability. Yet, aside from three studies [18, 19, 20] incorporating CHO ingestion strategies within laboratory‐based durability protocols, and despite emerging evidence from field‐based road racing simulations [21], the physiological mechanisms underpinning interindividual variability in durability are not well‐characterized.

With the progressive depletion of muscle and/or liver glycogen stores, it is plausible that decreased CHO substrate availability may contribute to the reduction in power output at intensity domain transitions during prolonged endurance exercise. To address the finite capacity of muscle glycogen stores, the consumption of CHO during exercise can enhance cycling performance through sustained exogenous CHO availability via the maintenance of plasma glucose concentrations and oxidation rates [6], liver glycogen sparing [22] and possibly muscle glycogen [23], and/or central facilitation of motor drive [24]. From a durability standpoint, maintaining exogenous CHO availability during prolonged exercise may be an important pre‐requisite to attenuating fatigue‐induced shifts in intensity domain transitions. Nonetheless, the extent to which graded CHO doses differentially preserve the power‐duration relationship remains unknown. Higher CHO ingestion rates up to 120 g·h^−1^ have been shown to increase exogenous CHO oxidation beyond that achievable at 60 or 90 g·h^−1^ [25], yet whether this translates to a dose‐dependent influence on durability has not been investigated.

With this in mind, the aim of our study was to examine the effects of graded CHO ingestion on durability of the CP following 3 h of moderate‐intensity cycling. We hypothesized that moderate‐intensity exercise would reduce the power output at the heavy‐to‐severe intensity transition, and that higher CHO ingestion rates would attenuate this decline.

## 2 Methods

### 2.1 Participants

Sixteen trained cyclists and triathletes (15 males, 1 female) were recruited to take part in this investigation (mean ± SD: age 35 ± 9 years, stature: 177 ± 9 cm, body mass: 76.8 ± 10.9 kg, lactate threshold: 179 ± 34 W, power output at 4 mmol·L^−1^: 255 ± 20 W, V̇O_2max_: 51.8 ± 6.8 mL·kg^−1^·min^−1^). Participants were free of illness and musculoskeletal injury, had no known cardiovascular or metabolic diseases, self‐reported training volume of > 5 h·week^−1^, and had a V̇O_2max_ of > 45 mL·kg^−1^·min^−1^. The female participant was premenopausal and was naturally menstruating. All participants completed a physical activity readiness questionnaire (PAR‐Q) and provided written informed consent. This study adhered to the standards outlined in the updated revision of the Declaration of Helsinki and received ethical approval from Liverpool John Moores University's Research Ethics Committee (24/SPS/003).

### 2.2 Experimental Overview

**FIGURE 1:**
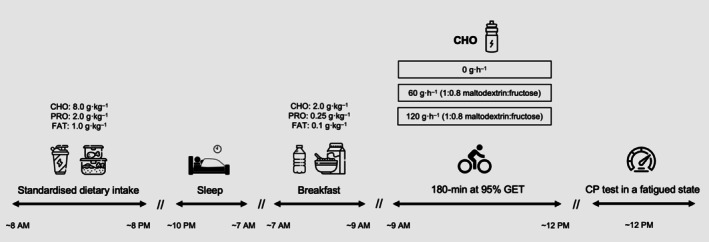
Schematic overview of the protocol adopted in each experimental trial (visits 3–5). Following the provision of a high‐CHO diet, subjects consumed a CHO‐rich breakfast before commencing a 180‐min steady‐state cycle in the moderate intensity domain, during which they consumed 0, 60, and 120 g·h^−1^ of a CHO drink mix, followed by a 3‐min critical power test in a fatigued state.

This study involved five laboratory visits, adopting a counterbalanced, randomized crossover design, with two characterization trials and three experimental trials, during which participants were provided with either 0 (water only), 60 or 120 g·h^−1^ of a CHO drink (Figure 1). A full counterbalancing approach was used, generating six possible permutations of the three conditions. These six sequences were each assigned to three participants, resulting in a fully counterbalanced sample of 18 participants. Following participant withdrawal (*n* = 2), the final sample consisted of 16 participants. The first visit was used to establish the baseline physiological characteristics of the participants and perform a familiarization session for the 3‐min all‐out CP test. The second visit (test day) was the 3‐min CP test performed in a non‐fatigued state. Visits 3–5 were the experimental trials consisting of 180 min of steady‐state cycling in the moderate intensity domain (at 95% of the estimated gas exchange threshold [GET]), followed by the 3‐min CP test performed in a fatigued state. Participants were provided with a standardized diet for the 24 h preceding each experimental trial, consisting of 8.0 g·kg^−1^ CHO, 2.0 g·kg^−1^ protein and 1.0 g·kg^−1^ fat. All food items were pre‐packaged and individually weighed to ensure precise dietary control. The characterization trials were separated by a minimum of 48 h, and experimental conditions were completed at least 6 days apart. All exercise tests were performed using the same cycle ergometer (Lode Excalibur Sport, Lode B.V., Groningen, Netherlands) and all expired gas analyses were taken with the same computerized metabolic cart following calibration according to manufacturer instructions (Vyntus CPX, Vyaire Medical, Höchberg, Germany).

### 2.3 Preliminary Testing

Participants arrived in the laboratory approximately 2.5 h after consuming a CHO‐rich breakfast (1.5 g·kg^−1^ CHO and 0.2 g·kg^−1^ protein) and having refrained from caffeine, vigorous exercise, and alcohol for 24 h prior. Before beginning the session, body mass and stature were recorded, and a resting capillary blood sample was collected via finger prick (Biosen C‐Line, EFK Diagnostics, Cardiff, UK) to measure lactate and glucose concentrations. Participants first completed a stepwise incremental protocol on an electrically braked cycle ergometer to identify individual lactate thresholds, followed by a ramp incremental test to determine V̇O_2max_ and the GET, which was subsequently used to set the intensity for the experimental trials. The step test commenced at 90 W (60 W for females), and the intensity increased by 30 W every 4 min thereafter until blood lactate reached a concentration of > 4 mmol·L^−1^, where the test terminated. A capillary blood lactate sample was collected from the fingertip at the end of each stage. Heart rate (Polar H10, Polar Electro Oy, Kempele, Finland), rating of perceived exertion (RPE) (Borg 6–20 scale), and expired gas were monitored throughout.

Following a 15‐min rest period, participants started the ramp test at 80 W, with the workload increasing by 5 W every 10 s (30 W·min^−1^) until volitional exhaustion or cadence dropped below 70 rpm. V̇O_2max_ was defined as the highest 30‐s V̇O_2_ rolling average recorded during the test, and peak power output (PPO) as the highest 30‐s mean power. GET was determined from the ramp incremental test using the V‐slope method (i.e., the first disproportionate increase in carbon dioxide output (V̇CO_2_) relative to V̇O_2_, and an increase in the ventilatory equivalent for O2 (V̇_E_/V̇O_2_) with no increase in the ventilatory equivalent for CO_2_ (V̇_E_/V̇CO_2_)), described by Beaver et al. [26]. To account for the lag in the V̇O_2_ response during the incremental test, two‐thirds of the ramp rate (i.e., 2/3 of 30 W = 20 W) was deducted from the power output at GET [27]. The GET and V̇O_2max_ were then used to apply the individual linear factor for each participant and normalize the fixed resistances for the CP tests.

Participants then rested for 30 min before commencing a familiarization test to the CP test, described below.

### 2.4 Critical Power Test Performed in a Non‐Fatigued State

The resistance for the CP test was applied using the linear (cadence‐dependent) mode of the ergometer and was calculated as linear factor = power output/preferred cadence^2^, where the power was 50% Δ (i.e., GET +50% of the difference between the GET and the ramp test PPO) and preferred cadence was the average cadence adopted by the participants in the ramp test (86 ± 6 rpm). The linear factor was 0.037 ± 0.006 (range 0.026–0.044) W/rpm^2^. Participants performed a 15‐min warm‐up at 120 W (90 W for females) and then the test began with a 3‐min baseline period at 20 W, where participants were allowed to pedal freely. During this period, participants were again reminded of the protocol to ensure the all‐out nature of the test and the criteria required for a valid 3‐min CP effort was understood. This was repeated for all CP tests in a fatigued state. A 10 s countdown was given before the CP test, during which participants were asked to increase cadence to ~110–120 rpm. Participants were instructed to pedal maximally from the start to finish and avoid pacing (i.e., reach peak power output as quickly as possible and maintain the cadence as high as possible throughout the test). Strong verbal encouragement was provided from the onset during all CP tests. Participants were not aware of the elapsed time and power output being sustained, with only cadence being displayed on‐screen. Researchers were also blinded to power output, with CP and work done above CP (W*′*, in kJ) for non‐fatigued and fatigued tests only being calculated after each participant had completed all experimental trials. CP was calculated as the average power output over the last 30 s of the test (end‐test power [EP]) and W*′* was defined as the total amount of work above EP (WEP) [28]. The 3‐min test in a non‐fatigued state established a control value for CP and W*′*.

### 2.5 Pre‐Experimental Controls

For the 48 h preceding each experimental trial, participants reduced their training volume and intensity. In the 24 h prior to each trial, participants refrained from any form of exercise and alcohol consumption and consumed a standardized, prepackaged high CHO diet containing exactly 8.0 g·kg^−1^ CHO, 2.0 g·kg^−1^ protein and 1.0 g·kg^−1^ fat. Caffeine consumption was permitted only before midday on the day before each trial and was prohibited thereafter until the completion of the trial the following day. Participants were also provided with a prepackaged CHO‐rich breakfast (2.0 g·kg^−1^ CHO, 0.25 g·kg^−1^ protein and 0.1 g·kg^−1^ fat) to be consumed ~2–2.5 h before the commencement of the 180‐min steady‐state cycle.

### 2.6 180‐Min Steady‐State Cycle and Critical Power Test in a Fatigued State

On the morning of the main experimental trials, participants reported to the laboratory at ~09:00 having consumed the same standardized CHO‐rich breakfast as described above. Participants completed a 5‐min warm‐up at 100 W and immediately began the 180‐min steady state cycle at 95% GET. This ventilatory marker was chosen as it has been previously used to demarcate the boundary between moderate‐ and heavy‐intensity exercise [29]. Expired gas was collected for a 4‐min period every 30 min to calculate whole body substrate utilization, total energy expenditure, gross efficiency and cycling economy. Gross efficiency was calculated from measures of V̇O_2_ and V̇CO_2_ averaged over the last 2 min of each 30‐min period, and power output at trial intensity. Gross efficiency (%) was defined as work rate/energy expenditure × 100, where energy expenditure was calculated using the formula of Jeukendrup and Wallis [30] for moderate‐ to high‐intensity exercise. Cycling economy was expressed as the oxygen cost of cycling (mL·min^−1^·W^−1^) from steady‐state V̇O_2_ during the prolonged cycle. Gastrointestinal (GI) symptoms (nausea, regurgitation, fullness, cramps, flatulence and urge to defecate) were recorded at 30‐min intervals using a 0–10 visual analog scale (0 = no discomfort and 10 = unbearable discomfort) [31]. Participants were instructed to complete the 10‐point GI scale as follows: 1 to 4 indicated mild GI symptoms (i.e., not substantial enough to interfere with exercise), 5 to 9 indicated severe GI symptoms (i.e., enough to interfere with exercise), and 10 indicated extreme GI symptoms warranting exercise cessation. The cumulative and peak scores were calculated for each symptom in each condition.

Upon completion of the 3‐h ride, participants were permitted a toilet break and the CP test in a fatigued state commenced ~3–5 min after finishing the submaximal cycle. Participants started the CP test with the same 3‐min baseline period at 20 W, during which they were again reminded of the importance of performing an all‐out effort from start to finish. The test protocol was replicated as described above.

### 2.7 Carbohydrate Feeding

The CHO drink was prepared by a member of the research team in the morning of each trial. Participants ingested a total of 1.35 L of a CHO beverage (150 mL every 20 min), containing either 180 or 360 g of unflavoured, multiple‐transportable CHO in a ratio of 1:0.8 of maltodextrin to fructose (Peak Supps, Bridgend, UK) to deliver CHO at an average rate of either 60 g·h^−1^ (20 g every 20 min, ~13.3% wt/vol CHO solution) or 120 g·h^−1^ (40 g every 20 min, ~26.7% wt/vol CHO solution). This ratio was chosen based on previous work demonstrating improved rates of oxidation and gastrointestinal comfort [29, 32]. The CHO beverage did not contain electrolytes or other ingredients beyond the maltodextrin‐fructose mixture described above. A fixed fluid volume across conditions was chosen for ecological validity. In the water only trial, participants drank 150 mL of water every 20 min to replicate the pattern of ingestion from the CHO mix in the other trials. Participants were allowed to drink water ad libitum throughout the exercise, and fluid intake was monitored in all experimental conditions. Body mass before and after the 180‐min cycle was measured to examine differences in fluid loss between trials.

### 2.8 Estimates of Whole Body Substrate Oxidation and Energy Expenditure

Rates of whole body CHO and fat oxidation (g·min^−1^) were measured at 30‐min intervals during the prolonged cycle with the equations of Jeukendrup and Wallis [30] for moderate to high intensity exercise. Hourly and total energy expenditure for each trial was estimated assuming an energy yield of 17.57 kJ and 39.33 kJ for 1 g of CHO and fat, respectively.

### 2.9 Statistical Analysis

All statistical analyses were performed with SPSS Statistics version 30 (IBM, United States). CP, W*′*, total whole body CHO and fat oxidation, and total energy expenditure were analyzed by one‐way repeated measures ANOVAs. Differences in mean CHO and fat oxidation, hour by hour energy expenditure, RER, RPE, heart rate, relative intensity, gross efficiency, cycling economy, and blood metabolites over time (30–180 min) and between conditions were analyzed by two‐way repeated measures ANOVAs. Sphericity was assessed using Mauchly's test, and when violated, the Greenhouse–Geisser correction was applied. Where a significant main effect was found, pairwise comparisons were conducted using Bonferroni‐adjusted post hoc tests. Paired mean differences are presented with 95% confidence intervals, and effect sizes expressed as Cohen's *d*, interpreted as small (0.2), moderate (0.5), and large (> 0.8). Due to non‐normally distributed data, Friedman's ANOVA was used to assess differences in mean cumulative and peak GI symptom scores, with pairwise comparisons analyzed with Wilcoxon's signed ranks test where a significant main effect was found. All figures were produced using GraphPad Prism 6.07 (GraphPad Software LLC, San Diego, CA 92108 USA). Unless stated otherwise, data in texts, figures, and tables are presented as means ± SD, with *p* < 0.05 denoting statistical significance.

## 3 Results

### 3.1 Characterization Trial

Mean V̇O_2max_ measured in the preliminary trial was 3.93 ± 0.40 L·min^−1^, corresponding to a mean PPO of 372 ± 33 W. Mean power at GET obtained from the ramp test was 178 ± 15 W, and therefore a power output of 169 ± 14 W was adopted during the prolonged cycle. The intensity of the trial amounted to 24 ± 3 kJ·kg^−1^ of total work done over the 180‐min steady‐state cycle.

### 3.2 CHO Feeding Ameliorates the Deterioration of Critical Power in a Dose Dependent Manner but Not W*′*

**FIGURE 2:**
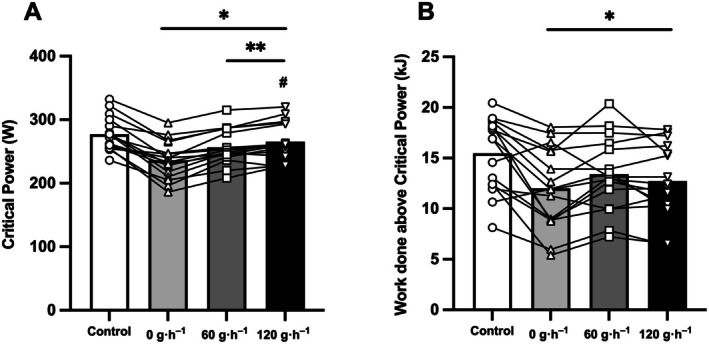
(A) End‐test power in a non‐fatigued (Control) and fatigued state following the ingestion of CHO at a rate of 0, 60 and 120 g·h^−1^. (B) Work done above end‐test power in a non‐fatigued (Control) and fatigued state under the same conditions. *Significantly different from Control. **Significantly different from 0 g·h^−1^. ^#^Significantly different from 60 g·h^−1^. All *p* < 0.05. Bars represent group means, with individual responses shown.

CP in a non‐fatigued state was significantly reduced in all experimental conditions (trial effect, *p* < 0.001), meaning CP in the 0 g·h^−1^ (236 ± 30 W), 60 g·h^−1^ (257 ± 28 W), and 120 g·h^−1^ (266 ± 29 W) trials was lower than Control (277 ± 27 W) (all *p* < 0.05; Figure 2A). However, the decline in CP was attenuated with increasing CHO intake, such that CP in the 60 g·h^−1^ trial was higher than 0 g·h^−1^ (mean difference [MD]: 20 W, *p* < 0.001, *d* = 1.92), and higher in 120 g·h^−1^ compared to 60 g·h^−1^ (MD: 9 W, *p* = 0.006, *d* = 1.01). This corresponded to a relative reduction in CP of 14.7% ± 7.1% in the 0 g·h^−1^, 7.4% ± 4.7% in the 60 g·h^−1^, and 4.2% ± 3.7% in 120 g·h^−1^ trials versus the non‐fatigued state. The % V̇O_2max_ attained during the 3‐min CP test also differed significantly between trials (*p* < 0.001). Specifically, % V̇O_2max_ was lower in the 0 g·h^−1^ (90.1% ± 3.4%) condition compared with the non‐fatigued state (97.7% ± 2.2%, *p* < 0.001), whereas no difference was observed between either CHO condition (60 g·h^−1^: 94.3% ± 3.7%, *p* = 0.050; 120 g·h^−1^: 96.7% ± 2.6%, *p* = 1.000) and the non‐fatigued state. Furthermore, % V̇O_2max_ achieved in the 3‐min CP test was progressively higher with increasing CHO ingestion, being greater in 60 g·h^−1^ versus 0 g·h^−1^ (*p* = 0.025), and in 120 g·h^−1^ versus 60 g·h^−1^ (*p* = 0.036). Control‐CP occurred at 51% ± 7% of the difference between GET and PPO.

W*′* also significantly declined following the prolonged exercise bout (trial effect, *p* < 0.001) independent of CHO feeding compared to a non‐fatigued state (Control‐W*′*: 15.5 ± 3.6 kJ), but this reduction was similar between 0 g·h^−1^ (12.0 ± 4.0 kJ), 60 g·h^−1^ (13.41 ± 3.7 kJ), and 120 g·h^−1^ (12.7 ± 3.6 kJ) (all *p* > 0.05; Figure 2B). The magnitude of the decline in W*′* was 22.0 ± 20.1, 12.6 ± 16.5, and 17.8% ± 12.9% in 0, 60, and 120 g·h^−1^ trials, respectively.

### 3.3 Physiological, Metabolic and Perceptual Responses to Exercise

Absolute oxygen uptake, heart rate and relative intensity (expressed as a percentage of V̇O_2max_) all increased during exercise (time effects, *p* = 0.025, *p* < 0.001 and *p* = 0.014, respectively) (Table 1), with no significant differences between trials (condition effect, all *p* > 0.05) and no condition × time interaction (all *p* > 0.05).

**TABLE 1:** Heart rate, RPE, absolute oxygen consumption, relative intensity and energy expenditure during 180‐min steady state cycling.

	Time (min)					
	30	60	90	120	150	180
Heart rate, beats·min^−1^						
0 g·h^−1^	127 ± 15	128 ± 14	129 ± 16	133 ± 14^a,b,c^	135 ± 15^b^	140 ± 16^a,b,c,d,e^
60 g·h^−1^	130 ± 12	130 ± 12	129 ± 14	131 ± 13	134 ± 13	136 ± 14^b,c,d^
120 g·h^−1^	129 ± 14	131 ± 13*	132 ± 14^a^	135 ± 15^a^	135 ± 16	139 ± 15^a^
RPE, AU						
0 g·h^−1^	10 ± 1	11 ± 1^a^	12 ± 1^a,b^	13 ± 2^a,b,c^	15 ± 2^a,b,c,d^	16 ± 2^a,b,c,d,e^
60 g·h^−1^	10 ± 2	12 ± 2^a^	13 ± 2^a,b^	13 ± 1^a,b^	14 ± 2^a,b,^ *	14 ± 2^a,b,c,d,^ *
120 g·h^−1^	10 ± 2	11 ± 2^a^	12 ± 1^a^	13 ± 1^a^	13 ± 2^a,b,c,^ *	14 ± 2^a,b,c,d,^ *
V̇O_2_, L·min^−1^						
0 g·h^−1^	2.36 ± 0.20	2.40 ± 0.20	2.40 ± 0.20	2.43 ± 0.22	2.43 ± 0.23	2.49 ± 0.26
60 g·h^−1^	2.36 ± 0.27	2.41 ± 0.28	2.41 ± 0.27	2.42 ± 0.27	2.47 ± 0.31	2.47 ± 0.34
120 g·h^−1^	2.37 ± 0.24	2.41 ± 0.30	2.40 ± 0.30	2.41 ± 0.25	2.41 ± 0.28	2.42 ± 0.29
Relative Intensity, % V̇O_2max_						
0 g·h^−1^	60 ± 6	61 ± 5	61 ± 5	62 ± 4	62 ± 5	63 ± 4
60 g·h^−1^	60 ± 8	61 ± 7	62 ± 7	62 ± 7	63 ± 6	63 ± 7
120 g·h^−1^	60 ± 5	61 ± 7	61 ± 6	61 ± 5	61 ± 5	61 ± 5
Energy Expenditure, kJ·min^−1^						
0 g·h^−1^	50.6 ± 3.7	51.5 ± 5.5	51.6 ± 5.7	51.5 ± 6.9	51.6 ± 6.5	51.2 ± 7.5
60 g·h^−1^	51.1 ± 4.4	51.5 ± 5.9	51.9 ± 6.2	51.6 ± 6.2	51.5 ± 7.0	51.3 ± 6.2
120 g·h^−1^	51.9 ± 4.0	51.3 ± 4.9	51.2 ± 6.1	51.0 ± 6.1	50.5 ± 5.2	50.5 ± 5.7

*Note:* Data are presented as means ± SD. AU, arbitrary units; RPE, rating of perceived exertion; V̇O_2_, oxygen consumption. a–e denotes *p* < 0.05 vs. ^a^30, ^b^60, ^c^90, ^d^120, and ^e^150 min, respectively.

* Significant difference from 0 g·h^−1^, *p* < 0.05.

**FIGURE 3:**
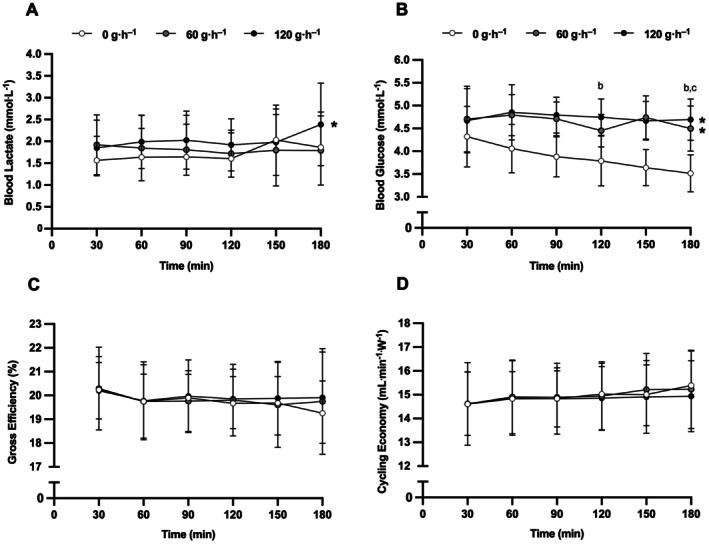
Blood lactate (A), blood glucose (B), gross efficiency (C), and cycling economy (D) during the 180‐min cycle in the 0, 60, and 120 g·h^−1^ trials. Data are means ± SD for *n* = 16 trained cyclists and triathletes. a–e denotes *p* < 0.05 versus ^a^30, ^b^60, ^c^90, ^d^120, and ^e^150 min, respectively. *Significant difference from 0 g·h^−1^, *p* < 0.05.

Blood lactate remained unchanged across the 180‐min cycle (time effect, *p* = 0.139), whereas blood glucose progressively decreased over time (time effect, *p* = 0.006) (Figure 3). Overall, blood lactate and blood glucose concentrations differed significantly between conditions (condition effect, *p* = 0.005 and *p* < 0.001, respectively), with a significant condition × time interaction for glucose (*p* = 0.002) but not lactate (*p* = 0.123). Pairwise comparisons showed no significant differences between 60 and 120 g·h^−1^ trials for both lactate and glucose (*p* = 0.080 and *p* = 1.000, respectively); however, lactate concentrations were higher in 120 g·h^−1^ compared with 0 g·h^−1^ (*p* = 0.004), while glucose concentrations were more elevated in both 60 and 120 g·h^−1^ trials compared with 0 g·h^−1^ (both *p* < 0.001). Simple main effect analyses for blood glucose revealed that this difference in both CHO conditions compared with 0 g·h^−1^ occurred from 60 min onwards (all *p* ≤ 0.002), with no differences between 60 and 120 g·h^−1^ at any timepoint (all *p* > 0.05).

RPE progressively increased during exercise (time effect, *p* < 0.001) and was significantly higher in the 0 g·h^−1^ trial compared with the 120 g·h^−1^ trial (*p* = 0.023) (Table 1). A significant interaction was also observed (*p* < 0.001), with simple main effects analyses showing higher RPE in the water only trial compared with both CHO feeding trials from 150 min onwards (150 min: *p* = 0.011 and *p* = 0.010 for 60 and 120 g·h^−1^ trials, respectively; 180 min: *p* < 0.001 for both CHO conditions).

Gross efficiency did not change during the trial (time effect, *p* = 0.155), whereas the oxygen cost of cycling increased throughout the 3 h (time effect, *p* = 0.009), with no differences between trials for either variable (condition effect, *p* = 0.790 and *p* = 0.799, respectively).

### 3.4 CHO Feeding Alters Whole Body Substrate Metabolism

**FIGURE 4:**
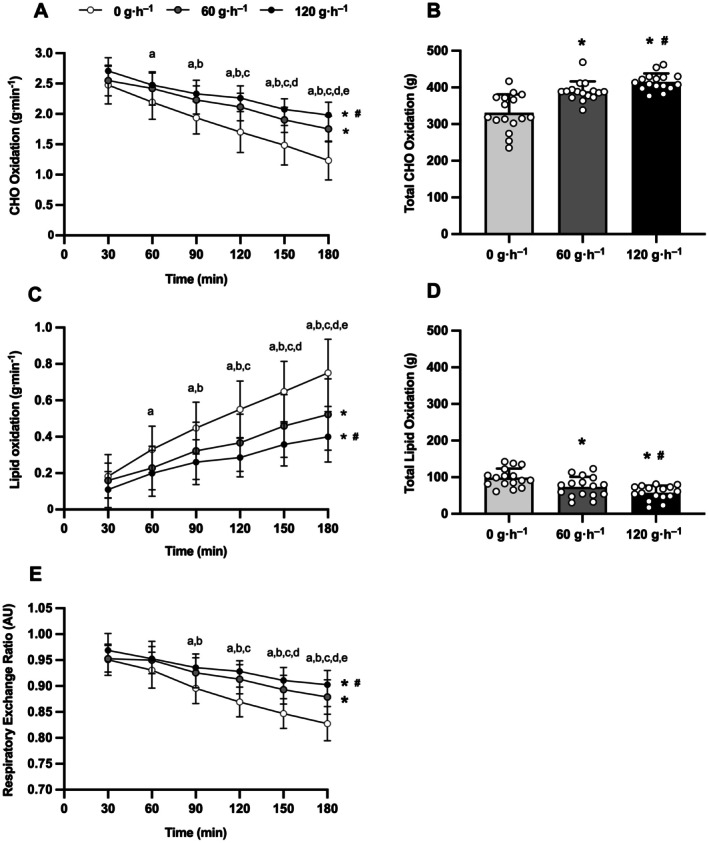
Rates of whole body CHO oxidation during exercise (A), total CHO oxidation (B), rates of whole body fat oxidation during exercise (C), total fat oxidation (D), and respiratory exchange ratio [RER] during exercise in the 0, 60, and 120 g·h^−1^ trials. Data are means ± SD for *n* = 16 trained cyclists and triathletes. a–e denotes *p* < 0.05 vs. ^a^30, ^b^60, ^c^90, ^d^120, and ^e^150 min, respectively. *Significant difference from 0 g·h^−1^, *p* < 0.05. ^#^Significant difference from 60 g·h^−1^, *p* < 0.05.

Rates of whole body CHO oxidation progressively decreased during exercise (time effect, *p* < 0.001) and this was accompanied by a progressive increase in lipid oxidation (time effect, *p* < 0.001) and a decline in RER (time effect, *p* < 0.001). With the graded doses of CHO ingestion, rates of whole body CHO (*p* < 0.001) and lipid utilization (*p* < 0.001), total CHO (*p* < 0.001) and lipid oxidation (*p* < 0.001), and RER (*p* < 0.001) during exercise were all significantly different between conditions (Figure 4, A–E, respectively). Specifically, rate of CHO oxidation (mean ± SD: 1.84 ± 0.28, 2.16 ± 0.15, 2.31 ± 0.14 g·min^−1^), rate of lipid oxidation (mean ± SD: 0.55 ± 0.14, 0.41 ± 0.16, 0.32 ± 0.11 g·min^−1^), total CHO oxidation (mean ± SD: 331 ± 51, 389 ± 28, 415 ± 23 g), total lipid oxidation (mean ± SD: 99 ± 25, 73 ± 28, 57 ± 20 g) and RER (mean ± SD: 0.86 ± 0.03, 0.90 ± 0.02, 0.92 ± 0.02) all displayed significant pairwise differences (mean values reported for 0, 60 and 120 g·h^−1^, respectively) between trials (all *p* ≤ 0.021), meaning 120 g·h^−1^ > 60 g·h^−1^ > 0 g·h^−1^ for rates of whole body CHO oxidation (MD: 0.14 g·min^−1^, *d* = 1.03; MD: 0.33 g·min^−1^, *d* = 1.16), total CHO oxidation (MD: 26 g, *d* = 1.05; MD: 58 g, *d* = 1.16) and RER (MD: 0.02, *d* = 0.83; MD: 0.04, *d* = 1.34), and 0 g·h^−1^ > 60 g·h^−1^ > 120 g·h^−1^ for rates of whole body lipid oxidation (MD: −0.09 g·min^−1^, *d* = −0.77; MD: −0.14 g·min^−1^, *d* = −0.92) and total lipid oxidation (MD: −16 g, *d* = −0.78; MD: −25 g, *d* = −0.91). All previous comparisons are reported for 120 versus 60 g·h^−1^, and 60 g·h^−1^ versus 0 g·h^−1^, respectively.

**FIGURE 5:**
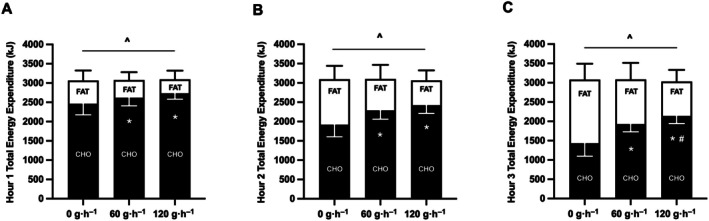
Total energy expenditure in the first (A), second (B), and third (C) hours of prolonged endurance cycling. ^Significant main effect between carbohydrate and fat contributions to total energy expenditure (interaction, *p* < 0.05). *Significant different from 0 g·h^−1^, *p* < 0.05. ^#^Significant difference from 60 g·h^−1^, *p* < 0.05.

**FIGURE 6:**
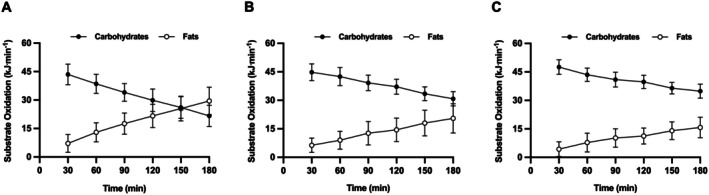
Rates of energy provision from carbohydrate and fat oxidation during the 0 g·h^−1^ (A), 60 g·h^−1^ (B), and 120 g·h^−1^ (C) trials.

Total exercise energy expenditure did not increase over time (*p* = 0.891) and was not different between conditions (*p* = 0.870). When the data were examined on an hour‐by‐hour basis (Figure 5), the contribution of CHO toward total energy expenditure was significantly greater than fat (substrate effect, *p* < 0.05 for hours 1, 2 and 3) and furthermore, CHO contributed a greater energy yield in the 60 and 120 g·h^−1^ trials compared with the 0 g·h^−1^ trial in all time periods (all *p* < 0.05). During the third hour of exercise, the energy yield derived from CHO was also greater in the 120 g·h^−1^ trial compared with the 60 g·h^−1^ trial (*p* = 0.008). The metabolic crossover point (i.e., the time course during exercise at which fat is the greatest contributor to total energy expenditure) occurred at approximately 150 min into the exercise, whereas both 60 g·h^−1^ (approaching unity at 180 min) and 120 g·h^−1^ prevented the occurrence of fat taking over as the primary contributor to total energy expenditure (Figure 6).

### 3.5 Increasing Rates of Drink‐Based CHO Feeding Do Not Alter Gastrointestinal Discomfort During Exercise

**FIGURE 7:**
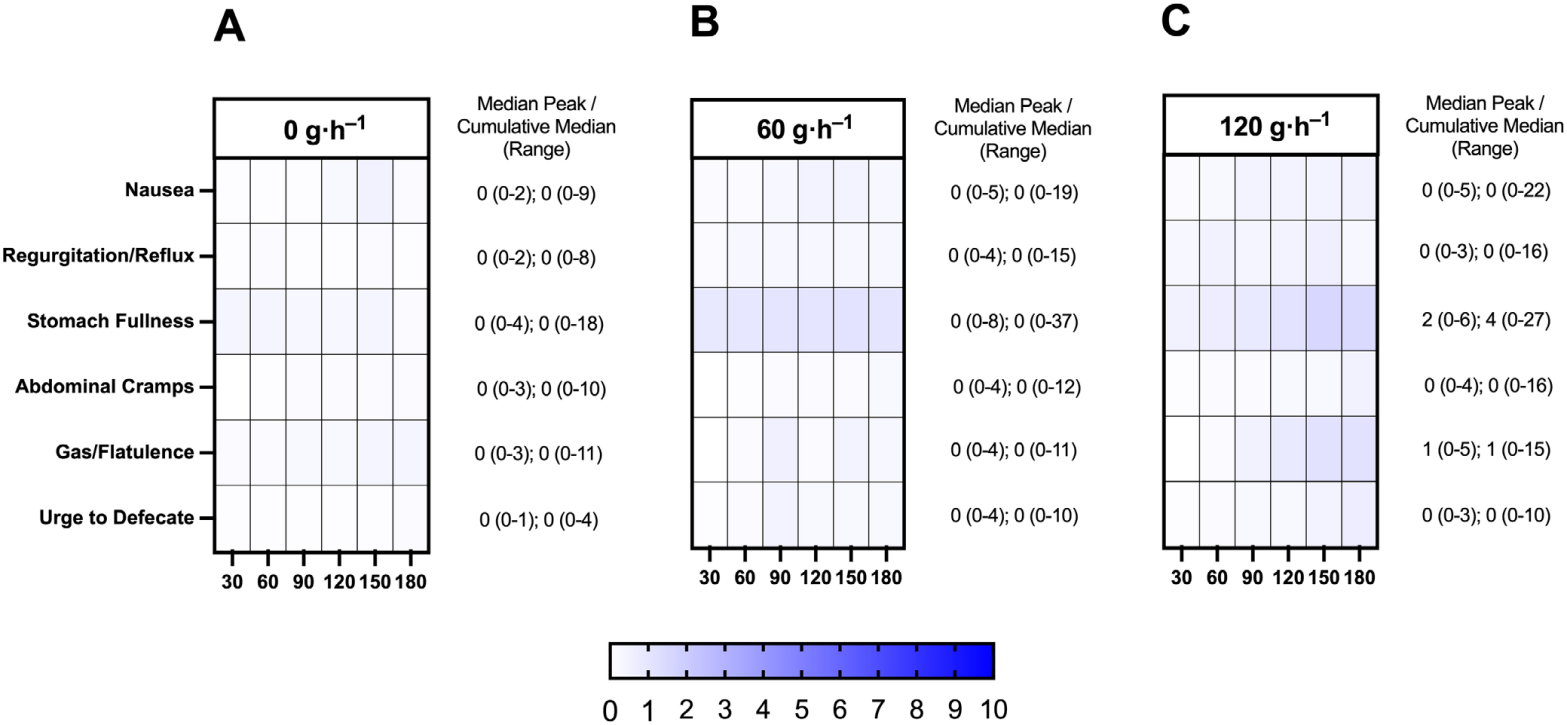
Gastrointestinal symptom prevalence during moderate intensity cycling in the 0 g·h^−1^ (A), 60 g·h^−1^ (B), and 120 g·h^−1^ (C) trials. Data are means ± SD for *n* = 16 trained cyclists and triathletes.

The incidence of moderate or severe (≥ 4) GI symptoms was negligible across all conditions (Figure 7), with only 4 participants reporting scores ≥ 4 (but below 8) at any given timepoint for any given symptom in the 60 and 120 g·h^−1^ conditions. Cumulative GI symptom scores differed significantly between conditions (*p* = 0.044; between CHO doses 6 [0–56], 13 [0–78], 18 [0–81] for 0, 60 and 120 g·h^−1^, respectively), with post hoc Wilcoxon's signed rank tests revealing higher scores in 120 g·h^−1^ compared with water only (*p* = 0.005), but no differences between 60 and 0 g·h^−1^ nor 120 and 60 g·h^−1^ (*p* > 0.05). Peak scores for regurgitation (*p* = 0.037), stomach fullness (*p* = 0.028) and abdominal cramps (*p* = 0.015) all varied between conditions. Specifically, regurgitation (MD: 0.6 AU, *p* = 0.026, *d* = 0.65), stomach fullness (MD: 1.1 AU, *p* = 0.019, *d* = 0.71) and abdominal cramps (MD: 0.4 AU, *p* = 0.034, *d* = 0.61) were all significantly higher at 120 g·h^−1^ compared with 0 g·h^−1^, with no differences between CHO conditions nor between 60 and 0 g·h^−1^. In contrast, peak scores for nausea, flatulence and urge to defecate did not differ significantly between conditions (all *p* > 0.05). There were no differences in total fluid intake between conditions (0 g·h^−1^: 1868 ± 398 mL, 60 g·h^−1^: 1849 ± 544 mL, 120 g·h^−1^: 1946 ± 539 mL; *p* = 0.573).

## 4 Discussion

To our knowledge, this is the first study to investigate the influence of graded CHO ingestion on the dynamics of the power‐duration relationship at the heavy‐to‐severe transition (i.e., CP) following prolonged endurance exercise in the moderate intensity domain. In the present cohort of trained road cyclists and triathletes, 3 h of steady‐state cycling exercise reduced CP by ~15%, indicating a significant deteriorative shift at the heavy‐to‐severe boundary and impaired durability in the absence of CHO ingestion during exercise. In line with our hypothesis, we report for the first time that graded CHO ingestion attenuated the decline in CP in a dose‐dependent manner, whereby the preservation of CP was greatest when athletes consumed CHO at a rate of 120 g·h^−1^. Of note, even the highest CHO ingestion rate was not sufficient to fully prevent the reduction in CP, suggesting that durability is multifactorial and, although related, cannot be explained by exogenous CHO availability alone. In contrast, W*′* decreased following prolonged exercise but was unaffected by CHO ingestion. Finally, whole‐body CHO oxidation increased progressively with CHO ingestion, indicating that greater exogenous CHO availability may have improved exercise capacity in the fatigued 3‐min CP test, as evidenced by the greater % V̇O_2max_ attained when 120 g·h^−1^ of CHO was consumed. Collectively, these data suggest that higher CHO doses may confer a metabolic and performance advantage by attenuating fatigue‐induced reductions in CP and promoting the stability of the power‐duration relationship.

CHO ingestion at a rate of 120 g·h^−1^ did not negate the reduction in CP observed after 3 h of moderate‐intensity cycling. This finding is not necessarily surprising given the duration of the exercise bout and amount of mechanical work accumulated for trained cyclists and triathletes. Similarly, Dudley‐Rode et al. [18] found that CHO ingestion at 60 g·h^−1^ limited, but did not abolish, the reduction at the moderate‐to‐heavy transition (from ~6% to ~3%) after 150 min of moderate‐intensity cycling (90% VT_1_). In contrast, Clark et al. [19] reported that 60 g·h^−1^ was sufficient to retain CP after a ~9% reduction following 2 h of heavy‐intensity exercise. This discrepancy should be interpreted in light of the substantially longer protocol duration, which may point to distinct physiological mechanisms underpinning the sensitivity of intensity domain boundaries to fatigue. In the absence of CHO ingestion, and considering the longer exercise bout, we report a~15% decline in CP, which is comparable with previous studies showing reductions of ~5%–10% at the moderate‐to‐heavy transition [13, 14, 16, 17, 18] and 8%–11% at the heavy‐to‐severe transition [19, 33, 34] following prolonged endurance exercise. Moreover, the difference in CP between both CHO conditions was small. Specifically, there was a ~7% and ~4% fall in CP when consuming 60 and 120 g·h^−1^, respectively, compared with Control. Given that ingesting 120 g·h^−1^ did not fully prevent the reduction in CP, it remains unclear whether ingestion rates closer to current CHO guidelines (i.e., 90 g·h^−1^) [35, 36] would elicit comparable preservation of the heavy‐to‐severe boundary during prolonged moderate‐intensity exercise.

A notable finding of the present study is that CP deteriorated following prolonged exercise without concomitant changes in physiological determinants of performance or metabolic responses (oxygen cost, gross efficiency, relative intensity, blood lactate, total energy expenditure), which indicates a dissociation between performance‐related durability (i.e., changes in the power‐duration curve) and physiological resilience. This dissociation suggests that the fatigue‐induced downward shift in CP cannot be fully explained by measurable changes in underlying physiological responses at this exercise intensity, and that whole‐body CHO utilization may be a primary driver of CP preservation. Although not directly assessed, greater liver glycogen sparing [22] and a small but meaningful attenuation of muscle glycogen utilization [23] with higher CHO ingestion rates possibly contributed to greater preservation of CP. The absence of differences in oxygen cost and gross efficiency may be partly explained by the exercise intensity employed (~60%–65% V̇O_2max_), where the substrate shift between conditions is smaller and total metabolic flux is lower, making any differences in efficiency unlikely to be detectable. At race intensities (i.e., closer to CP or critical speed), and when consuming 120 g·h^−1^ of CHO, the greater ATP yield per liter of O_2_ from CHO compared with fat oxidation becomes mechanistically meaningful and detectable [25]. Importantly, with the higher CHO ingestion rates, participants were able to attain a progressively higher % V̇O_2max_ in the fatigued 3‐min CP tests (~90%, ~94% and ~97% with 0, 60 and 120 g·h^−1^, respectively), suggesting that 120 g·h^−1^ of CHO preserved the capacity to attain exercise intensities close to V̇O_2max_ during the all‐out efforts in a fatigued state. Despite the absence of differences in traditional physiological markers, some participants reported RPE values of 17–18 toward the end of the 3 h cycle in the water only condition, and perception of effort was generally higher in the CHO conditions compared with 0 g·h^−1^. This suggests marked physiological strain and deterioration even if not captured by metabolic variables alone at this exercise intensity.

Recent data suggests that the boundaries between exercise intensity domains are not stable [13, 34], such that with increasing time and work done, a power output that commences in the moderate domain can drift into the heavy domain. During heavy‐intensity exercise, a delayed steady‐state in V̇O_2_ and blood lactate is attained, and muscle metabolic and ionic perturbations (PCr, Pi, H^+^) stabilize at elevated levels [37, 38, 39]. This results in a greater disturbance to muscle metabolic homeostasis than in moderate‐intensity exercise [12]. Increasing time spent within the heavy domain may lead to the development of peripheral fatigue [39], contributing to a reduction at the heavy‐to‐severe intensity transition, as reported herein. Supporting this idea, Gallo et al. [14] demonstrated that it takes on average~139 min of moderate‐intensity cycling at 90% VT_1_ for the moderate‐to‐heavy transition to decrease by 5%. Given that the fatiguing exercise bout was performed at a higher relative intensity (95% GET), it is conceivable that 3 h of moderate‐intensity cycling would provoke comparable deterioration of the moderate‐to‐heavy transition (in the region of 8%–15%) in the absence of CHO ingestion. Further investigations are required to quantify the dynamics of the power‐duration relationship following more prolonged exercise protocols.

Contrary to CP, CHO ingestion did not ameliorate the decline in W*′*. Defined as the curvature‐constant of the power‐duration relationship, W*′* represents a fixed amount of external work that can be performed during exercise above CP before exhaustion occurs. Contemporary research suggests W*′* is a complex, multifactorial parameter determined by disproportional changes in indices of substrate level phosphorylation (e.g., muscle [PCr], [Gly], [Cr], [La^−^]), progressive loss of muscular efficiency in all muscle fiber types, and greater V̇O_2_ slow component amplitude [39]. A reduction in W*′* following prolonged endurance exercise has been associated with greater muscle glycogen depletion in some studies [19] but not in others [33]. Without muscle glycogen data to verify this, our findings indicate prolonged moderate‐intensity exercise affected this finite work capacity uniformly regardless of exogenous CHO availability (evidenced by higher RER, a greater rate of CHO oxidation, and higher blood glucose when CHO was ingested). This observation is consistent with Clark et al. [19], who reported that CHO ingestion at 60 g·h^−1^ did not prevent the deterioration of W*′*. In their study, participants retained ~36% of their resting muscle glycogen concentrations following 2 h of heavy‐intensity exercise. Following prolonged exercise, reductions in W*′* may be partly attributed to the selective depletion of subcellular glycogen pools. Glycogen depletion in all three spatial compartments (intermyofibrillar, intramyofibrillar and subsarcolemmal) is likely substantial following 3 h of moderate‐intensity exercise, though the rate and extent of that depletion depend on exercise intensity and duration, muscle fiber type recruitment, and subcellular location [40, 41, 42]. Importantly, depletion of intramyofibrillar glycogen impairs contractile function via a decrease in sarcoplasmic reticulum calcium release rate [43], which may contribute to compromised exercise capacity at intensities above CP and thereby reduce W*′*.

From an applied perspective, tolerability of high CHO ingestion rates is an important consideration when determining the feasibility of nutritional strategies during prolonged endurance exercise. Despite higher cumulative GI scores in 120 g·h^−1^ compared with the water only condition, the incidence of GI symptoms across all experimental trials was generally mild (≤ 4) and mean scores did not differ between both CHO conditions. These findings suggest that ingestion rates of 120 g·h^−1^, whilst not entirely symptom‐neutral, can be well‐tolerated by trained cyclists and triathletes. Historically, the incidence of GI discomfort among athletes participating in endurance events can be high [44], and higher CHO ingestion rates (> 90 g·h^−1^) may increase the likelihood of experiencing such issues [45, 46]. However, when CHO is provided using a multiple‐source formulation of glucose‐ and fructose‐based CHO, studies have shown that superior exogenous CHO oxidation rates can be achieved with trivial GI symptomatology [29, 47], making the minimal GI disturbance observed in the present study unsurprising. Our findings are in line with recent evidence suggesting that ingestion rates of 120 g·h^−1^ are well‐tolerated during prolonged exercise in trained cyclists [29, 32], further supporting the feasibility of this nutritional strategy in an applied context.

Our study used the 3‐min all‐out test to expeditiously estimate CP in a non‐fatigued condition and immediately following 3 h of cycling in the moderate‐intensity domain. The 3‐min CP test uses a fixed linear factor to set the resistance based on the power output at 50% of the difference between GET and V̇O_2max_, and individual self‐selected cadence. Despite its reliability in a fatigued condition being established elsewhere [33], the linear factor derived from a resting state may not have fully accounted for fatigue‐induced alterations in the abovementioned physiological parameters after 3 h of exercise. Anecdotally, we found that participants with a linear factor at the lower end of the range were better able to maintain a cadence closer to their preferred cadence during the final 30 s of the test, which could have slightly inflated their CP. Consequently, it is possible that the magnitude of deterioration at the heavy‐to‐severe boundary was underestimated. The dynamics of the power‐duration relationship at the heavy‐to‐severe transition are difficult to ascertain under fatigued conditions, as traditional CP testing requires multiple exhaustive trials performed across separate visits. Within the context of exercise metabolism studies employing crossover designs and prolonged exercise protocols, such an approach would not have been feasible. The moderate‐to‐heavy intensity transition is an important parameter in training prescription and load monitoring [48], and the methodological challenges of assessing CP under fatigued conditions may have further directed research attention toward this boundary.

### 4.1 Perspectives

From a conceptual standpoint, the present findings provide novel insights into how graded CHO ingestion modulates the dynamics of the power‐duration relationship, with an intake of 120 g·h^−1^ conferring a performance advantage over 60 g·h^−1^ in limiting the downward shift at the heavy‐to‐severe intensity boundary. These findings carry important practical implications for both training and competition. From a training perspective, it is not uncommon for trained cyclists to perform high‐intensity intervals positioned later in a prolonged training session, once a substantial amount of mechanical work has already been accumulated. As demonstrated herein, a fatigued‐induced reduction in CP means that training zones derived from rested values do not accurately reflect an athlete's physiological boundaries in a fatigued state, potentially causing prescribed training intensities to reside in a higher domain than intended. Increasing CHO ingestion during exercise up to 120 g·h^−1^ may help preserve the power‐duration curve, allowing athletes to stay within the desired training zones for longer and perform subsequent efforts at power outputs closer to their fresh parameters. This not only improves training quality but may also translate to greater chronic adaptations. Furthermore, in race scenarios, the ability to resist the decline in physiological determinants of performance (i.e., exercise economy/efficiency, fractional utilization at defined thresholds) is crucial to avoid (or at least delay) the transition into higher intensity domains, and CHO ingestion rates up to 120 g·h^−1^ represent an effective nutrition strategy to achieve that.

## 5 Conclusion

In conclusion, the present investigation demonstrated prolonged moderate‐intensity cycling significantly reduced the power output at the heavy‐to‐severe intensity transition. CHO ingestion limited, but not prevented, the deterioration in CP in a dose‐dependent manner, with ingestion rates of 120 g·h^−1^ attenuating the decline in power output to a greater extent than 60 g·h^−1^. In contrast, the reduction in W*′* was comparable across conditions and was not influenced by CHO ingestion. These data highlight the importance of exogenous CHO availability in retaining the parameters of the power‐duration relationship and improving durability.

## Funding

This work was supported by the Portuguese Foundation for Science and Technology, 2023.00585.BD.

## Conflicts of Interest

This work was funded by the Portuguese Foundation for Science and Technology (FCT) through the individual doctoral grant 2023.00585.BD for the first author. Nutrition products for the experimental trials were provided by Peak Supps, Bridgend, UK.

## Data Availability

The data that support the findings of this study are available from the corresponding author upon reasonable request.
